# Dysparathyroidism: A Clinical Window

**DOI:** 10.4103/0256-4947.72282

**Published:** 2010

**Authors:** Nandini Chakrabarti, Chandan Chattopadhyay

**Affiliations:** a From the Department of General Medicine, NRS Medical College and Hospital, Kolkata, India; b From the Department of Pharmacology, KPC Medical College and Hospital Jadavpur, Kolkata, India

**Figure 1:**
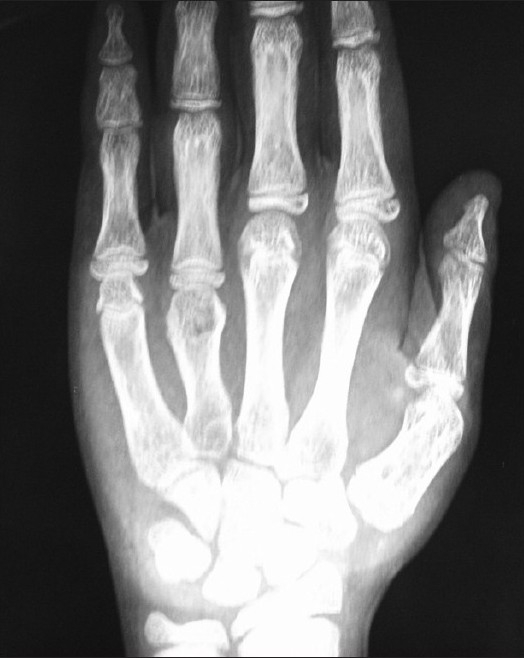
Photograph shows X-ray of hand AP view with short fourth metacarpal bone.

**Figure 2:**
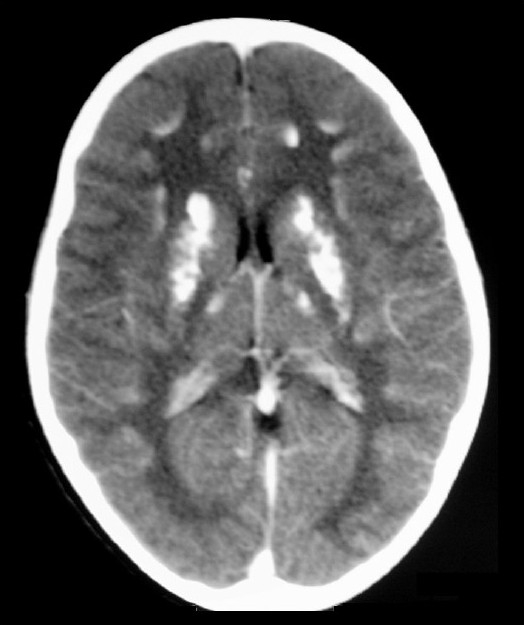
Photograph shows CT Scan of brain (plain) revealing bilateral basal ganglia calcification.

**Figure 3:**
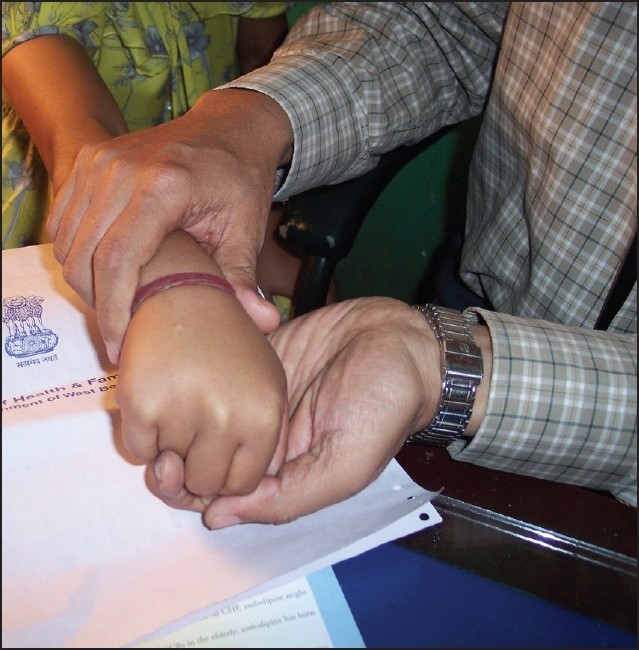
Photograph shows short ring finger of right hand.

**Figure 4:**
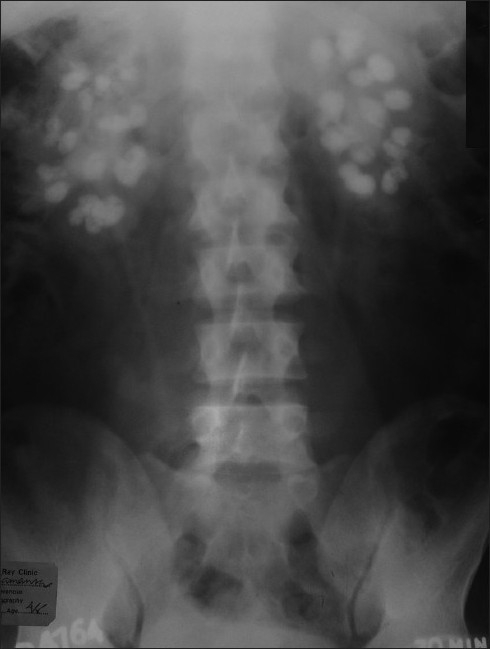
Photograph shows straight X-ray of abdomen with bilateral extensive nephrocalcinosis.

Calcifications in various tissues are a feature of both hyper- and hypocalcemic states. A 13-year-old girl presented with recurrent episodes of generalized tonic-clonic seizures for the previous three months. She was in the sixth standard with a history of learning difficulties. There was also a history of delayed milestones of development. On examination, the patient had a height of 123 cm (<3rd percentile), with an upper segment of 63 centimeters, weight 24 kg (<3rd percentile) and normal facies. There were bilateral short ring fingers (**Figures 1 and 2**), and short 3rd and 4th toes. Neurological examination showed a low IQ rating. Complete hemogram, urea, creatinine, liver function tests, chest x-ray, serum sodium, potassium and USG abdomen were normal. The EEG showed marked sharp wave activity while the CT scan of the brain revealed bilateral basal ganglia as well as parietal lobe calcifications (**Figure 3**). Serum calcium, phosphate and magnesium were 6.4 mg/dL (normal, 8-10 mg/dL), 7 mg/dL (normal, 2.5-5 mg/dL) and 2.5 mg/dL, respectively. The serum 25-hydroxy cholecalciferol level was 47 ng/mL (normal, 15-80 ng/mL) while serum PTH level was 450 pg/mL (normal, 15-65 pg/mL). X-ray of the kidney showed multiple bilateral stones (**Figure 4**).

This was a case of pseudohypoparathyroidism with features of Albright osteodystrophy and other trophic hormone resistance as well. Common sites involved are walls of the blood vessels, periarticular areas, skin, soft tissues and viscera, notably lung, heart, kidney, intestine and brain. Although phosphate is initially low in primary hyperparathyroidism, progressive renal impairment may later contribute to raise calcium phosphate product metabolism.^1^ The occurrence of visceral calcification in pseudohypoparathyroidism is a complicated issue. However, it has been postulated that visceral calcification exists as amorphous calcium phosphate.^2^ It has been documented that serum phosphate >8-9 mg/dL and/or calcium phosphate product >70 mg/dL, local tissue injury, increased parathormone level—all contribute to heterotopic calcification.^3^

Nephrocalcinosis refers to bilateral minute calcifications in the renal pyramids due to calcium deposition in the tubular epithelium. It may occur in the absence of stones and is often diagnosed incidentally as in our asymptomatic patient with unremitting hypertension. Basal ganglia calcification as seen in our patient may occur physiologically in 0.3% to 1.5% of the population. It may also be found in hyper- and pseudohypoparathyroidism.^4^

The presence of calcinosis cannot be used as an index of neurological impairment, but its extent and the presence of decreased perfusion demonstrated by PET may be useful in predicting symptomatic progression.^5^ Both hyper- and hypocalcemic states may give rise to metastatic calcifications. Incidental diagnosis of such calcifications should direct investigations towards parathyroid disorders.
